# Co-Immobilization of SpyTag-Cyclized Enzymes on a γPFD-SpyCatcher Hydrogel to Address Broad Specificity

**DOI:** 10.3390/gels12040348

**Published:** 2026-04-21

**Authors:** Ming-Yue Huang, Qing-Yi Su, Tao Wei, Fu-Xing Niu

**Affiliations:** 1 Guangxi Key Laboratory for Green Processing of Sugar Resources, Guangxi University of Science and Technology, Liuzhou 545006, China; 100002482@gxust.edu.cn (M.-Y.H.);; 2 Department of Bioengineering, College of Food Science, South China Agricultural University, Guangzhou 510640, China; 3 State Key Laboratory of Non-Food Biomass Energy Technology, Nanning 530000, China

**Keywords:** biosynthesis, SpyTag/SpyCatcher, nanoscaffold, enzyme promiscuity, coimmobilization

## Abstract

The broad substrate specificity of enzymes, while advantageous for catalytic diversity, often leads to undesired side reactions and reduced product yields in engineered metabolic pathways. To address this challenge, we developed a programmable protein scaffold based on a self-assembled γPFD-SpyCatcher hydrogel for the in vivo co-immobilization of SpyTag-cyclized cascade enzymes, enabling the co-immobilization of cascade enzymes in a spatially organized manner. Enzymes with broad substrate specificities were linearly fused with SpyTags, facilitating their spatial organization on the nanoscaffold within engineered *E. coli* to ensure directed catalytic flux. Using this strategy, the yields of pinene and caffeoyl-CoA were enhanced by 5.8-fold (reaching 94.5 mg/L) and 2.4-fold (reaching 78.6 mg/L), respectively, compared to free enzyme systems. This work establishes an effective approach to mitigate the limitations posed by broad enzyme specificity and demonstrates its potential for applications in synthetic biology and industrial biotechnology.

## 1. Introduction

The broad substrate specificity of enzyme-catalyzed reactions presents a major challenge in metabolic engineering. Although this characteristic offers a versatile platform for pathway design, it frequently results in precursor depletion, by-product accumulation, and reduced yields of target products in multi-enzyme cascades [1]. For instance, in monoterpene biosynthesis, farnesyl diphosphate synthase (IspA) not only produces the desired geranyl diphosphate (GPP) but also converts it further to farnesyl diphosphate (FPP), thereby diverting metabolic flux away from the intended products [2]. Similarly, in the aromatic amino acid pathways, enzymes such as tyrosine ammonia lyase (TAL) [3], 4-coumaroyl-CoA ligase (4CL) [4], and the widely recognized P450 enzymes—often described as “nature’s most versatile biological catalysts” [5,6]—exhibit broad substrate ranges, leading to complex product mixtures and inefficient carbon flux. Traditional strategies, such as enzyme fusion or scaffolding, often fail to fully mitigate the effects of such broad substrate specificity [7].

Recent advances in protein nanotechnology have enabled the spatial organization of enzymes to enhance catalytic efficiency [8]. However, most existing systems do not adequately address the issue of conformational flexibility, which is conducive to broad substrate catalysis. The SpyTag/SpyCatcher system, developed by Howarth and colleagues [9], is based on the internal isopeptide bond within the second immunoglobulin-like collagen adhesin (CnaB2) domain. Upon mixing, SpyCatcher and SpyTag spontaneously form a highly specific isopeptide bond between Lys31 on SpyCatcher and Asp117 on SpyTag. This system has been widely used for enzyme stabilization and immobilization [10,11,12]. To further enhance the thermal stability of enzymes, a SpyTag/SpyCatcher-mediated cyclization strategy was subsequently developed [13]. For example, Li et al. successfully improved the thermal stability of intracellular D-amino acid oxidase (RgDAAO) using this Spy cyclization system [14].

In our previous work, to address issues associated with high enzyme dispersion in cell-free synthesis systems, such as low local concentrations of target enzymes per unit volume and feedback inhibition by substrates, we combined the Spy system with the γPFD protein nanoscaffold. The γPFD nanoscaffold has been widely employed in enzyme immobilization studies [15]. Interestingly, we discovered that SpyCatcher could promote hydrogel formation by the nanoscaffold [16]. In contrast, previously reported methods for preparing hydrogels based on nanoscale scaffolds typically require additional chemical crosslinking or other treatments [17,18,19,20]. Building on these findings, we hypothesized that combining SpyTag-mediated enzyme cyclization with scaffold immobilization could impose a more rigid, linear architecture on the enzymatic assembly and thereby direct the substrate flux.

Here, we report the in vivo application of this comprehensive strategy. Given that the pinene and caffeyl coenzyme A synthesis pathways have a well-established research foundation in our research team and involve multiple enzymes with broad substrate catalytic properties, these will be the main focus of this study. By co-immobilizing SpyTag-cyclized enzymes on the γPFD-SpyCatcher hydrogel scaffold within engineered *E. coli*, we constructed artificial substrate channels that significantly enhanced product yield and selectivity. This approach provides a versatile and scalable platform for precision metabolic engineering.

## 2. Results and Discussion

### 2.1. In Vivo Assembly of γPFD-SpyCatcher Hydrogels and Its Impact on Cell Physiology

In our previous study, we confirmed that γPFD-SpyCatcher forms a hydrogel in vitro after being expressed in cells [16]. To further characterize this hydrogel, we evaluated its physical properties. Rheological analysis of cell lysates (Figure S1) revealed a continuous solid network with a storage modulus (G′) of approximately 1.44 Pa and a loss factor (tan δ) of 0.39, indicating the formation of a soft, elastic hydrogel (Table 1 and Figure S2). Hydrogels composed of natural biomacromolecules that offer improved ionic stability, biocompatibility, and ionic conductivity are in high demand for advanced food and biomedical applications, but their rational design remains challenging [21,22,23]. The protein nanoscale scaffold hydrogel mediated by SpyCatCher has provided a new direction for research in the field of hydrogels, and the characteristics of this type of hydrogel may have more potential development areas. Notably, under production conditions, the formation of the hydrogel did not substantially alter its macroscopic morphology. Although it remains unclear whether this hydrogel assembles into similar nanostructures inside living cells as it does in vitro, the observed in vitro properties suggest that comparable effects may occur in vivo—an aspect that warrants further investigation (More research needs to be conducted in the next).

**Figure 1:**
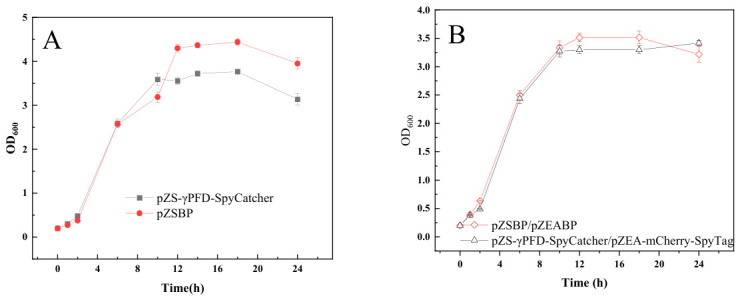
The influence of γPFD-SpyCatcher hydrogels on cell growth. (**A**) Express γPFD-SpyCatcher alone in *E. coli*. (**B**) The γPFD-SpyCatcher and mCherry-SpyTag were co-expressed in *E. coli*.

We next compared the growth curves of strains carrying different constructs: the pZSBP vector alone, pZS-γPFD-SpyCatcher, the dual-plasmid system pZSBP/pZEABP, and pZS-γPFD-SpyCatcher/pZEA-mCherry-SpyTag. As shown in Figure 1A, in vivo formation of the γPFD-SpyCatcher hydrogel significantly affected cell growth, resulting in an approximately 15% reduction in biomass. Figure 1B further demonstrates that the presence of two plasmids also imposes a negative effect on growth. pZSBP is the backbone vector of pZS-γPFD-SpyCatcher, and pZEABP is the backbone vector of pZEA-mCherry-spyTag. The comparison of the above growth conditions indicates that the in vivo expression of the nanoscale scaffold hydrogel and the co-localization of enzymes may impose certain burdens on the cells, thereby affecting their growth.

### 2.2. In Vivo Enzyme Immobilization

**Figure 2:**
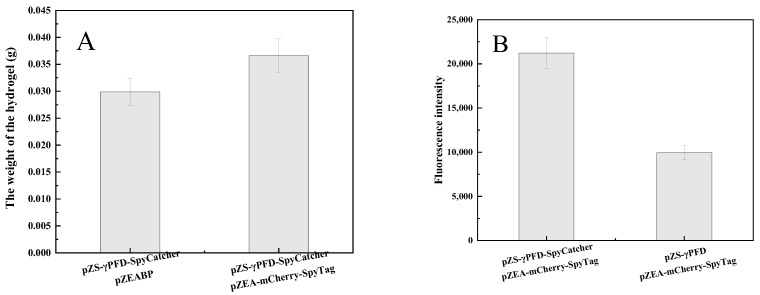
The weight of the hydrogel and the fluorescence intensity of mCherry loaded by γPFD-SpyCatcher in vivo. During the stationary phase, a certain volume of cell solution is taken, such that the product of the cell density value (OD_600_) and the volume (in mL) reaches the value of 4. (**A**) The weight of the hydrogel. (**B**) The fluorescence intensity of mCherry loaded by γPFD-SpyCatcher.

The loading capacity of enzymes immobilized on the γPFD-SpyCatcher nanoscaffold is a critical parameter for evaluating scaffold performance. To assess this, we used cultures of equal cell density co-expressing either pZS-γPFD-SpyCatcher/pZEABP (control) or pZS-γPFD-SpyCatcher/pZEA-mCherry-SpyTag. Following cell lysis, the mass of the resulting hydrogel was measured (Figure 2A). The results indicate that the loading capacity of the mCherry-SpyTag enzyme on the γPFD-SpyCatcher hydrogel reached approximately 220 mg per gram of nanoscaffold hydrogel (22 wt.%). However, although the rheological data in vitro confirmed the formation of the hydrogel, its in vivo structure has not yet been clearly characterized. Further in-depth studies can be conducted using cryo-electron microscopy in the future.

To evaluate the enzymatic activity after co-immobilization, bacterial cells of equal density were analyzed (Figure 2B). Notably, the fluorescence intensity of the immobilized mCherry increased by 2.1-fold compared to its free counterpart, indicating enhanced specific activity upon scaffold binding. The first report utilizing nanofibers as supports for enzyme immobilization was presented by Jia et al. [24], achieving a loading of only 1.4 wt.% and retaining merely 65% of the native enzyme activity. Although subsequent studies on related nanoscaffolds have been more extensive, the activity of immobilized enzymes has generally remained below 100% in most cases [25]. In contrast, our SpyCatcher-mediated nanoscaffold system demonstrates significant advantages, offering both superior enzyme loading capacity and a marked enhancement of fluorescence intensity (or enzymatic activity). However, although the fluorescence intensity of mCherry was quantitatively measured, the microscopic localization of this hydrogel in the body still needs to be further clarified in future studies.

### 2.3. Co-Immobilization of Cascade Enzymes for Enhanced Pinene Biosynthesis

**Figure 3:**
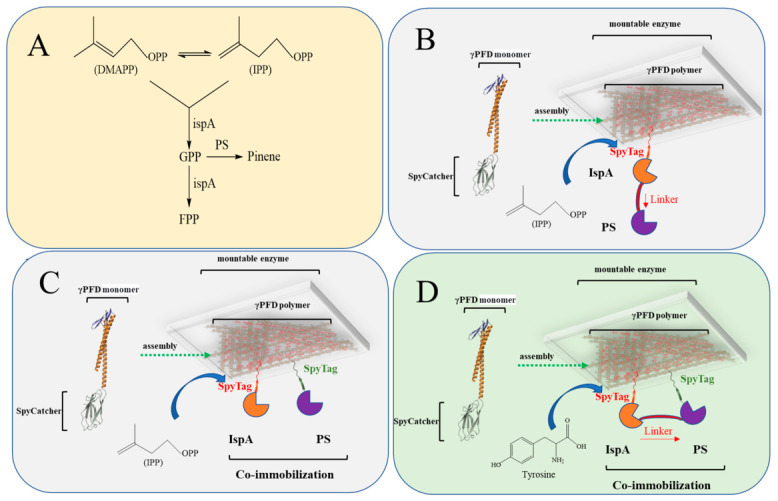
Schematic illustration of the γPFD-SpyCatcher hydrogel scaffold for the co-immobilization of SpyTag-cyclized IspA and PS. DMAPP: dimethylallyl diphosphate; IPP: isopentenyl diphosphate; FPP: farnesyl pyrophosphate. GPP: geranyl pyrophosphate; IspA: FPP synthase. (**A**) ispA and PS catalyze the synthesis of the pinene metabolic pathway. (**B**) The co-immobilization of γPFD-SpyCatcher with SpyTag-ispA-PS. (**C**) The co-immobilization of γPFD-SpyCatcher with SpyTag-ispA and SpyTag-PS. (**D**) The co-immobilization of γPFD-SpyCatcher with SpyTag-ispA-L-PS-SpyTag.

α-Pinene is a biologically active monoterpene that has been successfully synthesized in genetically engineered microorganisms by co-expressing IspA and pinene synthase (PS) [26,27] (Figure 3A). As shown in Figure 3A, IspA exhibits broad substrate specificity, catalyzing not only the conversion of IPP to GPP, the precursor for pinene formation by PS, but also the further conversion of GPP to FPP, thereby depleting the pinene precursor pool available for pinene synthesis. As summarized in Table 2A, when IspA and PS are overexpressed, the pinene yield reaches only 16.37 mg/L. The low yield can be attributed to three main factors: (1) α-Pinene exhibits high toxicity toward *E. coli* [27]; (2) the heterogeneous expression of PS in *E. coli* leads to enzyme dispersion and non-aggregation, reducing catalytic efficiency per unit volume; and (3) the broad substrate specificity of IspA results in precursor loss [2].

To address the challenge of broad substrate specificity in enzyme catalysis, we first attempted to co-localize the enzymes using a linker-bridging strategy. However, as shown in Table 2B, this approach did not lead to a significant increase in pinene yield. We therefore turned to an alternative method involving the co-immobilization of the cascade enzymes IspA and PS using SpyCatcher-mediated γPFD protein nanoscaffolds, with the aim of increasing local enzyme concentration (Table 2C). This co-immobilization strategy resulted in a substantial improvement, enhancing pinene yield by 3.6-fold.

To further optimize the system, we compared two immobilization approaches: direct co-immobilization of IspA and PS versus immobilization after linking them with a flexible peptide linker. The results indicated that although co-immobilization alone improved production, the yield was lower than that achieved with the pre-connected IspA-L-PS fusion. This difference may be attributed to the fact that the broad substrate specificity of IspA was not fully constrained, and efficient metabolic flux was not fully achieved. In previous studies, Dueber et al. developed synthetic protein scaffolds that enable modular recruitment of metabolic enzymes; however, their system was designed to optimize heterologous pathway flux rather than to specifically manage enzymes with broad substrate specificity [7]. Similarly, Zhang et al. employed EutM nanoscaffolds and the SpyTag/SpyCatcher system to co-immobilize two cascade enzymes for chiral amine synthesis, demonstrating superior performance over free enzymes [8]. Together, these reports suggest that conventional strategies exhibit limited efficacy in controlling enzymes with pronounced catalytic promiscuity.

Although the IspA-L-PS enzyme in the pinene synthesis pathway was successfully immobilized, its C-terminal region remained flexible, which may have prevented efficient “closed-loop” catalysis. To address this limitation, we constructed a cyclized enzyme variant using the SpyTag system (Figure 3D and Table 2E). As shown in Table 2E, the SpyTag-cyclized enzyme enabled more efficient catalytic flux, enhancing pinene yield by 3.9-fold. To further demonstrate the promoting effect of the nano-scaffold on the co-immobilized enzymes, we removed the γPFD-SpyCatcher from this system (Table 2G). The results showed that without the presence of the γPFD-SpyCatcher scaffold, the yield of pinene synthesis decreased significantly. To investigate whether the γPFD-SpyCatcher scaffold combined with SpyTag-cyclized enzymes promotes directional metabolic flux, we attempted to measure changes in GPP content before and after the reaction. However, due to the instability of FPP and the difficulty of its accurate quantification, we were unable to complete this analysis at the current stage. We further explored increasing the copy number of PS synthase, which raised the pinene titer to 94.52 mg/L, a 5.8-fold improvement over the initial yield. In previous work, we employed extensive engineering strategies—including heterologous enzyme mutagenesis, screening, and tunable intergenic regions (TIGR)—to improve pinene production, yet achieved only limited gains [27]. In contrast, the co-immobilization of SpyTag-cyclized enzymes on γPFD nanoscaffold hydrogels proved highly effective in minimizing off-target reactions caused by broad substrate specificity in the cascade pathway. This study demonstrates that such an integrated cyclization-and-scaffolding approach can effectively constrain enzyme promiscuity through spatial co-localization of cascade enzymes.

### 2.4. The Co-Immobilization of γPFD-SpyCatcher with SpyTag-Cyclized Enzymes for Caffeoyl-CoA Production

**Figure 4:**
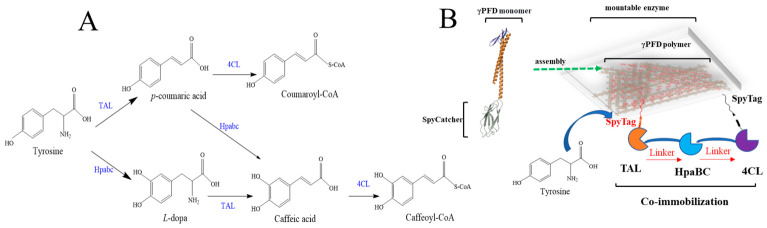
Schematic illustration of the γPFD-SpyCatcher hydrogel scaffold for the co-immobilization of SpyTag-cyclized HpaBC, TAL, and 4CL. Hpabc: p-hydroxyphenylacetate 3-hydroxylase; TAL: tyrosine ammonia lyase; 4CL: 4-coumarate CoA ligase. (**A**) HpaBC, TAL and 4CL catalyze the synthesis of tyrosine in the caffeoyl-CoA metabolic pathway. (**B**) The co-immobilization of γPFD-SpyCatcher with SpyTag- HpaBC-TAL-4CL.

To further evaluate the effect of spatial organization mediated by the SpyCatcher–γPFD nanoscaffold co-immobilized with SpyTag-cyclized cascade enzymes, we next targeted the caffeoyl-CoA biosynthesis pathway, in which tyrosine ammonia lyase (TAL), HpaBC, and 4-coumaroyl-CoA ligase (4CL) exhibit overlapping substrate specificities (Figure 4A). As illustrated in Figure 4A, TAL, HpaBC, and 4CL exhibit broad substrate ranges: TAL catalyzes not only the conversion of tyrosine to p-coumaric acid, but also the transformation of L-DOPA to caffeic acid; HpaBC hydroxylates tyrosine to L-DOPA and also converts p-coumaric acid to caffeic acid; 4CL activates both caffeic acid and p-coumaric acid to their corresponding CoA esters. Such catalytic promiscuity leads to inefficient carbon flux, substrate competition, and reduced overall catalytic efficiency. To address this, we constructed a SpyTag–HpaBC–TAL–4CL–SpyTag tandem expression module for co-immobilization on the γPFD–SpyCatcher scaffold (Figure 4B and Table 3B). Compared with the free enzyme system (Table 3A), the co-immobilized assembly doubled caffeoyl-CoA production and significantly reduced the accumulation of caffeic acid (Table 3B). This result further confirms that co-immobilization of SpyTag-cyclized enzymes on the γPFD nanoscaffold hydrogel effectively mitigates the challenges posed by enzyme promiscuity in metabolic cascades. Although other SpyCatcher-mediated scaffolding systems may support similar strategies, their performance requires further experimental validation.

Notably, L-DOPA readily oxidizes to dopaquinone, which exhibits a dark brown to black color, distinct from the yellowish-green appearance of caffeic acid (Supplementary Figure S4). Visual inspection of the fermentation broth indicated substantial residual L-DOPA in the co-immobilized system (Table 3B), likely due to the higher in vivo activity of HpaBC relative to TAL (Supplementary Figure S3). To better balance the reaction steps, we swapped the positions of TAL and HpaBC to construct a TAL-HpaBC-4CL cascade (Table 3C) This rearrangement led to considerable accumulation of caffeic acid (48.6 ± 3.1 mg/L) compared to the previous configuration (18.5 ± 2.6 mg/L in Table 3B), while caffeoyl-CoA titer increased modestly from 60.3 ± 3.5 mg/L to 78.6 ± 4.6 mg/L. This observation can be explained by the relative activities of the three enzymes in the cascade. When HpaBC is placed upstream of TAL (Table 3B), the high activity of HpaBC rapidly converts tyrosine to L-DOPA, but the subsequent conversion of L-DOPA to caffeic acid by TAL becomes rate-limiting, resulting in L-DOPA accumulation. In contrast, when TAL is placed upstream of HpaBC (Table 3C), the conversion of tyrosine to p-coumaric acid by TAL proceeds efficiently, and the downstream HpaBC then converts p-coumaric acid to caffeic acid. Because HpaBC exhibits higher catalytic activity toward p-coumaric acid than toward tyrosine (as shown in Supplementary Figure S3), the caffeic acid intermediate accumulates, yet the overall flux toward caffeoyl-CoA is enhanced due to the improved spatial proximity of the co-immobilized enzymes. Notably, this configuration also reduced L-DOPA accumulation, indicating that enzyme ordering within the co-immobilized complex influences metabolic flux distribution, and this finding is consistent with a spatial co-localization effect rather than a rigid, channeled tunnel.

Although increasing the copy number of 4CL might further enhance production, we decided against this approach due to constraints in plasmid carrying capacity and concerns regarding expression stability. Liquid chromatography analysis did not reveal significant coumaroyl-CoA production under the conditions tested, further supporting that the co-immobilization strategy effectively directs metabolic flux toward the target product.

### 2.5. Mechanistic Insights and Comparative Advantage

The SpyTag-cyclized enzymes, when immobilized on the γPFD hydrogel, adopt a constrained linear conformation that directs the flow of metabolic flux. This constrained architecture is distinct from prior scaffolding approaches, which primarily enhance local enzyme concentration but do not restrict conformational flexibility. However, these results only indicate that this system can effectively address the issue of the broad range of enzyme-catalyzed substrates, making the enzyme-linked reaction more directional. However, whether the application of this method has any impact on the kinetics of the enzyme reaction still requires further independent measurement and analysis.

Rheological measurements confirmed the formation of a soft, elastic hydrogel. The loss factor (tan δ = G″/G′) remained below 1, indicating its elastic-dominated behavior and moderate mechanical strength. This balanced viscoelastic profile suggests that the hydrogel can effectively prevent enzyme leakage while maintaining efficient mass transfer, thereby offering broad applicability. In addition, the observed decrease in shear viscosity with increasing time or shear rate reflects typical shear-thinning behavior, which is advantageous for practical handling and processing. Based on these findings, we propose that the soft, elastic nature of the SpyCatcher-mediated hydrogel contributes significantly to enhanced catalytic activity, while the engineered spatial organization helps enforce reaction specificity. Together, these features improve both the efficiency and selectivity of the enzymatic cascade. The potential of similar hydrogel systems warrants further investigation.

## 3. Conclusions

Here, we develop a modular protein hydrogel system based on γPFD-SpyCatcher for organizing SpyTag-cyclized multi-enzyme complexes. In this design, cascade enzymes are linked linearly and flanked by SpyTags, allowing them to be co-immobilized into the scaffold to achieve spatial proximity and enhance catalytic directionality. This architecture enhances catalytic directionality and alleviates inefficiencies caused by enzyme promiscuity. The presented method provides a versatile and scalable strategy for precision metabolic engineering, further accelerating the development of synthetic biology.

## 4. Materials and Methods

### 4.1. Strains and Plasmids

Plasmids and strains used in this study are listed in Table 4. *E. coli* BL21(DE3) was used for protein expression. The protein sequences used in this study are listed in Supplementary Table S1.

### 4.2. Construction of Plasmids

Codon-optimized SpyCatcher from *S. pyogenes*, γPFD from *Methanococcus jannaschii (M. jannaschii)* were synthesized by ANSHENGDA (Suzhou, China) and ligated into pZSBP with *Nhe*I and *Hin*dIII cleavage sites to obtain the pZS-γPFD-SpyCatcher. The codon-optimized SpyTag from *S. pyogenes*, pinene synthesis gene PS from *Agelas grandis (A. grandis)*, FPP synthesis gene *ispA* from *E. coli* W, 4-hydroxyphenylacetate 3 monooxygenase gene hpaB and hpaC with (GSG)_2_ Linker from *E. coli* W, tyrosine ammonia lyase gene tal from *Rhodotorula glutinis (R. glutinis)*, 4-coumaroyl-coenzyme A ligases gene 4CL from *Arabidopsis thaliana* (*A. thaliana)* and mCherry from *Discosoma* sp were synthesized by ANSHENGDA (Suzhou, China) and ligated into pZEABP, pZACBP and pZSBP with *Nhe*I and *Hin*dIII cleavage sites to obtain pZEA-ispA, pZAC-PS, pZEA-SpyTag- ispA, pZAC-SpyTag-PS, pZEA-SpyTag- TAL, pZAC- TAL-spyTag, pZS-4CL-SpyTag, pZEA-mCherry-SpyTag, pZEA-SpyTag- ispA -L-PS and pZEA-SpyTag- ispA -L-PS-SpyTag. pZEA-HpaBC, pZEA-SpyTag-HpaBC-TAL-4CL-SpyTag, pZEA-SpyTag- TAL- HpaBC-4CL-SpyTag and pZEA-SpyTag- TAL- HpaBC-2X4CL-SpyTag. The relevant protein sequences can be found in the attachment (Supplementary Table S1).

### 4.3. In Vivo Assessment of γPFD-SpyCatcher Hydrogel Effects on Cell Growth

A single colony was inoculated with LB medium and cultured at 37 °C, 200 rpm/min. This overnight seed culture was then used to inoculate 50 mL of fresh LB medium at a starting OD_600_ of 0.2, followed by incubation under the same conditions (37 °C, 200 rpm/min). Samples were collected every 2 h. The absorbance of OD_600_ was measured using a SynergyNeo2 multimode reader (SynergyNeo2, BioTek, Winooski, VT, USA). Prior to measurement, the bacterial culture was diluted as necessary to ensure that OD_600_ readings fell within the linear range of 0.2 to 0.8.

### 4.4. In Vivo Hydrogel Formation and Enzyme Immobilization

The γPFD-SpyCatcher fusion protein self-assembles into a hydrogel network upon expression in *E. coli.* Co-expression with SpyTag-fused enzymes results in the covalent immobilization of the enzymes via spontaneous SpyTag/SpyCatcher ligation inside the cells. Cells were harvested, lysed by sonication, and then the cell lysates were placed on ice and incubated with 4 mL of 500 mM ammonium sulfate for 4 h. After that, the insoluble hydrogel fraction was collected by centrifugation at 10,000× *g* for 10 min. The immobilization efficiency was quantified as the percentage of total enzyme activity (measured in the cell lysate) that was retained in the pelleted hydrogel fraction.

### 4.5. Loading Efficiency of Enzymes Immobilized In Vivo by γPFD-SpyCatcher

A single colony was inoculated with LB medium and cultured at 37 °C, 200 rpm/min. This seed culture was used to inoculate 50 mL of fresh LB medium at an initial OD_600_ of 0.2, followed by incubation under the same conditions. When cell growth reached the stationary phase, the OD_600_ of the culture was measured using a SynergyNeo2 multimode reader (SynergyNeo2, BioTek). A volume of culture (in mL) equivalent to an OD_600_ value of 4 was collected. The cells were collected by centrifugation at 10,000 rpm for 2 min at 4 °C, washed once with 0.1 M phosphate buffer (pH 7.0), and lysed using a high-pressure homogenizer (ANTUOSI ah-basic, Shouzhou, China) at 1500 bar.

The cell lysate was kept on ice and incubated with 4 mL of 500 mM ammonium sulfate for 4 h. After centrifugation at 8000× *g* for 5 min at 4 °C, the pelleted hydrogel was collected. The protein loading rate was calculated according to the following formula:

$$\frac{\mathrm{Hydrogel}2-\mathrm{Hydrogel}1}{\mathrm{Hydrogel}1}\times 100\%=\mathrm{Loading}\;\mathrm{Rate}\;\left(\%\right)$$

Hydrogel1: Hydrogel produced by co-expressing pZS-γPFD-SpyCatcher and the empty vector pZEABP. Hydrogel2: Hydrogel produced by co-expressing pZS-γPFD-SpyCatcher and pZEA-Enzyme-SpyTag.

### 4.6. Cell Growth and Production

For pinene production, SBMSN medium with 20% dodecane (trap pinene) was used, and an initial OD_600_ of 0.2 was set. After the fermentation process is completed, the dodecane on the surface of the fermentation liquid is collected for sample analysis. SBMSN medium containing sucrose 20 g/L, peptone 12 g/L, yeast extract 24 g/L, KH_2_PO_4_ 1.7 g/L, K_2_HPO_4_ 211.42 g/L, MgCl_2_·6H_2_O 1 g/L, ammonium oxalate 1.42 g/L, and Tween-80 2 g/L. (pH 7.0). For caffeoyl-CoA production, a fermentation medium was used, and an initial OD_600_ of 0.2 was set. The fermentation medium (pH 7.0) containing glucose 10 g/L, peptone 12 g/L, yeast extract 24, KH_2_PO_4_ 3 g/L, Na_2_HPO_4_·7H_2_O 13 g/L, NaCl 0.5 g/L, MgSO_4_·7H_2_O 0.24 g/L, NH_4_Cl 1 g/L, CaCl_2_ 0.1 g/L, proline 1 g/L, acidhydrolyzed casein 1 g/L. The main cultures were then incubated at 37 °C and 200 rpm/min until an OD_600_ reached 0.8. Then, the cultures were incubated at 30 °C and 200 rpm/min for 72 h.

### 4.7. Characterization of Hydrogel and Immobilized Enzymes

The viscoelastic properties, characterized by the storage and loss moduli (G’ and G’’), were evaluated as functions of frequency, shear stress, and time using a TA Instruments Discovery Hybrid Rheometer (New Castle, DE, USA) fitted with a 4 mm parallel plate. Measurements of the mechanical properties of γPFD-SpyCatcher were conducted at 25 °C under a strain of 2% and a reference frequency of 10 rad/s, employing both frequency- and time-sweep modes. Frequency sweeps were performed over a range of 0.1 to 100 Hz, while shear stress sweeps were carried out across a strain range of 0.1% to 100%.

### 4.8. Assay

OD_600_ was used for cell growth measurement. Pinene quantification was performed using gas chromatography coupled with flame ionization detection (GC-FID) (Techcomp GC7900, Techcomp Ltd., Shanghai, China) on a TM-5 capillary column (30 m × 0.32 mm × 0.50 μm). The injector temperature was maintained at 300 °C, with a constant carrier gas flow of 1 mL/min. The column oven temperature was initially held at 50 °C for 30 s, then programmed to rise at 4 °C/min to 70 °C, followed by a ramp of 25 °C/min to 240 °C. For analysis of DOPA, caffeic acid, and caffeoyl-CoA, high-performance liquid chromatography (HPLC) was employed using an Agilent HC-C18 column (5 μm, 4.6 × 250 mm). Separation was achieved with a mobile phase gradient of acetonitrile containing 0.1% trifluoroacetic acid (TFA), increasing from 10% to 70% over 20 min, followed by a return to 10%. Detection was carried out with a photodiode array detector (SPD-M20A) (Shimadzu Ltd., Kyoto, Japan) at wavelengths of 280 nm, 323 nm, and 344 nm, and compound concentrations were determined by reference to a standard curve generated from serial dilutions of a stock solution.

### 4.9. Statistical Analysis

Experiments were carried out in triplicate. The resulting data were averaged and presented as the mean ± standard deviation. Statistical analysis was performed using OriginPro (version 9.1), with one-way analysis of variance followed by Tukey’s test. A *p*-value of less than 0.05 was considered statistically significant.

## Figures and Tables

**Table 1:** SpyCatcher-mediated γPFD protein hydrogel rheological properties.

	Sample	γPFD-SpyCatcher
Character		
Structural Strength (G’)		continuous solid network structure (~1.44 Pa)
Viscous/Elastic Ratio (tan δ)		elastic samples (~0.39)
Zero-shear Viscosity (η_0_)		‘shear thinning’ type
Structural Stability		Stable
Yield Stress		medium-strength
Shear-thinning Behavior		Observed

**Table 2:** Pinene production using different combinations of IspA and PS.

Name	Strain	Expression Plasmid	Pinene (mg/L)
A	*E. coli* MEVI	pZEA-ispA, pZAC-PS	16.3 ± 1.1
B	*E. coli* MEVI	pZEA-ispA-L-PS	17.4 ± 1.2
C	*E. coli* MEVI	pZEA-SpyTag-ispA-L-PS, pZS-γPFD-SpyCatcher	58.5 ± 3.6
D	*E. coli* MEVI	pZEA-SpyTag-ispA, pZAC-SpyTag-PS, pZS-γPFD-SpyCatcher	21.4 ± 2.7
E	*E. coli* MEVI	pZEA-SpyTag-ispA-L-PS-SpyTag, pZS-γPFD-SpyCatcher	64.5 ± 4.3
F	*E. coli* MEVI	pZEA-SpyTag-ispA-L-2XPS-SpyTag, pZS-γPFD-SpyCatcher	94.5 ± 4.1
G	*E. coli* MEVI	pZEA-SpyTag-ispA-L-PS-SpyTag, pZSBP	15.4 ± 1.6

**Table 3:** Caffeoyl-CoA production using different combinations of HpaBC, TAL and 4CL.

Name	Strain	Expression Plasmid	Caffeic Acid (mg/L)	Caffeoyl-CoA (mg/L)
A	*E. coli* TYR	pZEA-HpaBC, pZAC-TAL pZS-4CL	44.2 ± 2.1	32.4 ± 2.8
B	*E. coli* TYR	pZEA-SpyTag-HpaBC-TAL-4CL-SpyTag, pZS-γPFD-SpyCatcher	18.5 ± 2.6	60.3 ± 3.5
C	*E. coli* TYR	pZEA-SpyTag-TAL- HpaBC-4CL-SpyTag, pZS-γPFD-SpyCatcher	48.6 ± 3.1	78.6 ± 4.6

**Table 4:** Strains and Plasmids used in this study.

Strains/Plasmids	Description	Source/Purpose
Strain		
*E. coli* DH5α	recA endA1 gyrA96 thi-1 relA1supE44 Δ(lacZYA-argF) U169 (Φ80lacZ ΔM15) hsdR17	Invitrogen
*E. coli* MEVI	CIChE strain from *E. coli* YZFP after integration of the mevalonate pathway	[27]
*E. coli* BW25113	lacIq rrnBT14ΔrhaBADLD7 hsdR514 ΔaraBADAH33 8ΔlacZWJ16	[28]
*E. coli* TYR	L-tyrosine producing strain, *E. coli* BW25113, ΔtyrR, ΔcsrA, ΔptsHI, Δcrr, P37-galP-P37-glk, Δzwf, ΔpheLA	[29]
Plasmid		
pZEABP	Constitutive expression vector, P37 promoter, pBR322 ori, Amp^r^	[30]
pZEA-mCherry-SpyTag	pZEABP fusion protein of mCherry (*Discosoma sp.*) to a SpyTag domain via a (GSG)_2_ linker.	This study
pZACBP	Constitutive expression vector, P37 promoter, Cm^r^, p15A ori	[30]
pZEA-ispA	pZEABP derivatives containing FPP synthase gene *ispA* from *E. coli* W	This study
pZAC-PS	pZACBP derivatives containing the codon-optimized pinene synthase gene *PS* from *Agelas grandis* (*A. grandis)*	This study
pZEA—ispA -L-PS	pZEABP derivatives containing FPP synthase gene *ispA* and pinene synthase gene PS with (GSG)_2_ linker	This study
pZEA-SpyTag- ispA -L-PS	pZEA-ispA-L-PS containing a SpyTag at N-terminal with (GSG)_2_ linker	This study
pZEA-SpyTag- ispA	pZEA-ispA containing a SpyTag at N-terminal with (GSG)_2_ linker	This study
pZAC-SpyTag-PS	pZAC-PS containing a SpyTag at N-terminal with (GSG)_2_ linker	This study
pZEA-SpyTag- ispA -L-PS-SpyTag	pZEA-SpyTag-ispA-L-PS containing a SpyTag at C-terminal with (GSG)_2_ linker	This study
pZEA-HpaBC	pZEABP derivatives containing 4-hydroxyphenylacetate 3 monooxygenase gene *hpaB* and *hpaC* with a (GSG)_2_ linker from *E. coli* W	This study
pZEA-SpyTag-HpaBC	pZEA-HpaBC containing a SpyTag at N-terminal	This study
pZAC-TAL	pZACBP derivatives containing the codon-optimized tyrosine ammonia lyase gene *tal* from *Rhodotorula glutinis (R. glutinis)*	This study
pZSBP	Constitutive expression vector, pBBR1 ori, P37 promoter, Kan^r^	[30]
pZS-4CL	pZSBP derivatives containing the codon optimized 4-coumaroyl-coenzyme A ligases gene *4CL* from *Arabidopsis thaliana* (*A. thaliana)*	This study
pZEA-SpyTag-HpaBC-TAL-4CL-SpyTag	pZEABP derivatives containing 3 monooxygenase gene hpaBC, tyrosine ammonia lyase gene *tal*, 4-coumaroyl-coenzyme A ligases gene *4CL* and two SpyTag at N-terminal and C-terminal respectively with (GSG)_2_ linker.	This study
pZEA-SpyTag- TAL- HpaBC-4CL-SpyTag	pZEABP derivatives containing tyrosine ammonia lyase gene *tal*,3 monooxygenase gene *hpaBC*, 4-coumaroyl-coenzyme A ligases gene *4CL* and two SpyTag at N-terminal and C-terminal respectively, with (GSG)_2_ linker.	This study
pZEA-SpyTag- TAL- HpaBC-2 × 4CL-SpyTag	pZEABP derivatives containing tyrosine ammonia lyase gene *tal*,3 monooxygenase gene *hpaBC*, two copies of 4-coumaroyl-coenzyme A ligases gene *4CL* and two SpyTag at N-terminal and C-terminal respectively, with (GSG)_2_ linker.	This study
pZS-γPFD-SpyCatcher	pZSBP derivatives containing the γPFD from *Methanococcus jannaschii* (*M. jannaschii*) and connected a SpyCatcher domain via a (GSG)_2_ linker	This study

## Data Availability

The dataset is available on request from the authors.

## Supplementary Materials

The following supporting information can be downloaded at: https://www.mdpi.com/article/10.3390/gels12040348/s1. Supplementary Table S1: Sequence of enzymes used in this study. Supplementary Figure S1 SpyCatcher-mediated γPFD nanoscaffold hydrogel. Supplementary Figure S2 SpyCatcher-mediated γPFD protein hydrogel viscoelastic properties. Supplementary Figure S3 HPLC chromatogram. Supplementary Figure S4 Color contrast during the fermentation process.
