# Supplementary material for: Co-Immobilization of SpyTag-Cyclized Enzymes on a γPFD-SpyCatcher Hydrogel to Address Broad Specificity
Source: Gels. 2026 Apr 21;12(4):348. doi: 10.3390/gels12040348 (PMC13116799; doi:10.3390/gels12040348)
Supplement: Supplementary file 1 [file gels-12-00348-s001.zip › gels-4218884-supplementary.pdf]

Co-Immobilization of SpyTag-Cyclized Enzymes on a γPFD-SpyCatcher Hydrogel to Address Broad Specificity

Ming-Yue Huang [1], Qing-Yi Su [1], Tao Wei [2] and Fu-Xing Niu [1,3,*]

[1] Guangxi Key Laboratory for Green Processing of Sugar Resources, Guangxi University of Science and Technology, Liuzhou 545006, China; 100002482@gxust.edu.cn (M.-Y.H.); 20250401031@stdmail.gxust.edu.cn (Q.-Y.S.)
[2] Department of Bioengineering, College of Food Science, South China Agricultural University, Guangzhou 510640, China; weitao@scau.edu.cn
[3] State Key Laboratory of Non-Food Biomass Energy Technology, Nanning 530000, China
* Correspondence: niufx3@gxust.edu.cn

Email:

Fu-Xing Niu: niufx3@gxust.edu.cn

Qing-Yi Su: 20250401031@stdmail.gxust.edu.cn

Tao Wei: weitao@scau.edu.cn

Ming-Yue Huang: 100002482@gxust.edu.cn

***Correspondence author:** Fu-Xing Niu: Guangxi Key Laboratory for Green Processing of Sugar Resources, Guangxi University of Science and Technology, Liuzhou 545006, China.

State Key Laboratory of Non-Food Biomass Energy Technology, Nanning 530000, China.

*Email address: 100002482@gxust.edu.cn

Supplementary Table S1 Sequence of enzymes used in this study

Name	Sequence
SpyCatcher	gdsathikfskrdedgkelagatmelrdssgktistwisdgqvkdfylypgkytfvetaapdgyevataitftvneqgqvtvng
SpyTag	mahivmvdaykpt
ɤPFD	mvnevidineavrayiaqieglraeigrldatiatlrqslatlkslktlgegktvlvpvgsiaqvemkvekmdkvvvsvgqnisaeleyeealkyiedeikklltfrlvleqaiaelyakiedliaeaqqtseeekaeeeeneekae
ispA	mdfpqqleacvkqanqalsrfiaplpfqntpvvetmqygallggkrlrpflvyatghmfgvstntldapaaavecihayslihddlpamddddlrrglptchvkfgeanailagdalqtlafsilsdadmpevsdrdrismiselasasgiagmcggqaldldaegkhvpldalerihrhktgaliraavrlgalsagdkgrralpvldkyaesiglafqvqddildvvgdtatlgkrqgadqqlgkstypallgleqarkkardliddarqslkqlaeqsldtsalealadyiiqrnk
PS	gpgslqrnglqqgtsnqrfgfhvgngitvtvlvavvertcnvecvffsadwnvniwlqqfpvqafdyvgdhrvnviqsvffrhtwvvfhitrngrgfftastvrfvatgvatqtqnsarqvvqfrrkvhflqnmirernihsqdrlqsadtmaarnftvfvvfikgrqipgrnpasllhvrvdvrfpclarigqgvtalslfsltghfihgvvnsniyafhvlwqainarrvpafhsggeqfqfvygaegvvhivqhgdqvtgfceaqtetavlrfksdaarkrivfnvtvsaeghfretgflppayqrfqltflkgledvkfqfrqfqeffglnirlsvfcilpenlnivtsfqtrqvsvptvfqhiadfaaqrgdrdflqclfqifggknfcfihdffarkcnqrrakqv*natdffvgldvfragelailtfecfedirghrvamqtqgtqaqsgavqvgydrltttpnavltpvrvyvvqsafdfifemtvdaqtfqgiydpqaldqvvqrahqltifqthavehvlyftdkffrtfqvgfflvcgrqrldhvvvpkvrvkithttthavvghngsgrhgdarsdrfatatrttrsfef

HpaBC	Mgsshhhhhhssglvprgshmasmahivmvdaykptkgsggsgmkpedfrastqrpftgeeylk<br>slqdgreiyiygervkdvtthpafrnaaasvaqlydalhkpemqdslcwntdtgsggythkffrvaksa<br>ddlrqqrdaiaewsrlsygwmgrtpdykaafgcalganpgfygqfeqnarnwytriqetglyfnhaiv<br>nppidrhlptdkvkdvyikleketdagiivsgakvvatnsalthynmvgfgsaqvmgenpdfalmfv<br>apmdadgvklisrasyemvagatgspydyplssrfdendailvmdnvlipwenvliyrdfdrcrrwt<br>meggfarmyplqacvrlavkldfitallkkslectgtlefrgvqadlgevvawrntfwalsdsmcseatp<br>wvngaylpdhaalqtyrvlapmayakikniiernvtsgliylpssardlnnpqidqylakyvrgsngm<br>dhvqrikilklmwdaigsefggrhelyeinysgsqdeirlqclrqaqssgnmdkmmamvdrclsey<br>dqngwtvphlhnnddinmldkllkgsggsgmqldeqrlrfrdamaslsaavniittegdtgqcgitata<br>vcsvtdtppslmvcinansamnpvfqgngklcvnvlnheqelmarhfagmtgmameerfslscwq<br>kgplaqpvlkgslaslegeirdvqaigthlvylveikniilsaeghgliyfkrrfhpvmlemeaai*
TAL	Maprptsqnqtrtcpttqvtqvdivekmlaaptdstleldgyslnlgdvvsaarkgrpvrvkdsdeirsk<br>idksveflrsqlsmsvygvttgfggsadtrtedaislqkallehqlcgvlpssfdsfrlgrglenslplevvr<br>gamtirvnsltrghsavrlvvlealtnflnhgitpivplrgtisasgdlsplsyiaaaisghpdskvhvvheg<br>kekilyareamalfnlepvvlgpkeglglvngtavsasmatlalhdahmlsllsqsltamtveamvgh<br>agsfhpflhdvtrphptqievagnirkllegsrfavhheeevkvkddegilrqdryplrtspqwlgplvs<br>dlihahavltieagqsttdnplidvenktshhggnfqaaavantmektrlglaqigklnftqltemlnag<br>mnrglpsclaaedpslsyhckgldiaaaaytselghlanpvtthvqpaemanqavnslalisarrttesn<br>dvlslllathlycvlqaidlraivfefkkqfgpaivslidqhfgsamtgsnlrdelvekvnktlakrleqtns<br>ydlvprwhdafsfaagtvvevlsstslslaavnawkvaaaesaisltrqvretfwsaastsspalsylsprt<br>qilyafvreelgvkarrgdvflgkqevtigsnvskiyeaiksgrinnvllkmla*

4CL	Mapqeqavsqvmekqsnnnnsdvifrsklpdiyipnhlslhdyifqnisefatkpclingptghvytysdvhvisrqiaanfhklgvnqndvvmlllpncpefvlsflaasfrgatataanpfftpaeiakqakasntkliitearyvdkikplqnddgvvivciddnesvpipegclrfteltqstteasevidsveispddvvalpyssgttglpkgvmlthkglvtsvaqqvdgenpnlyfhsddvilcvlpmfhiyalnsimlcglrvgaailimpkfeinlllleliqrckvtvapmvppivlaiakssetekydlssirvvksgaaplgkeledavnakfpnaklgqgygmteagpvlamslgfakepfpvksgacgtvvrnaemkivdpdtgdslsrnqpgeicirghqimkgylnnpaataetidkdgwlhtgdigliddddelfivdrlkelikykgfqvapaeleallighpditdvavvamkeeaagevpvafvvkskdselseddvkqfvskqvvfykrinkvfftesipkapsgkilrkdlraklangl*

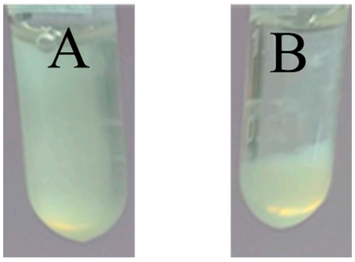

Supplementary Figure S1 SpyCatcher-mediated γPFD nanoscaffold hydrogel.

(A) Normal cells rupture without precipitating any water gel. (B) γPFD nanoscaffold protein hydrogel.

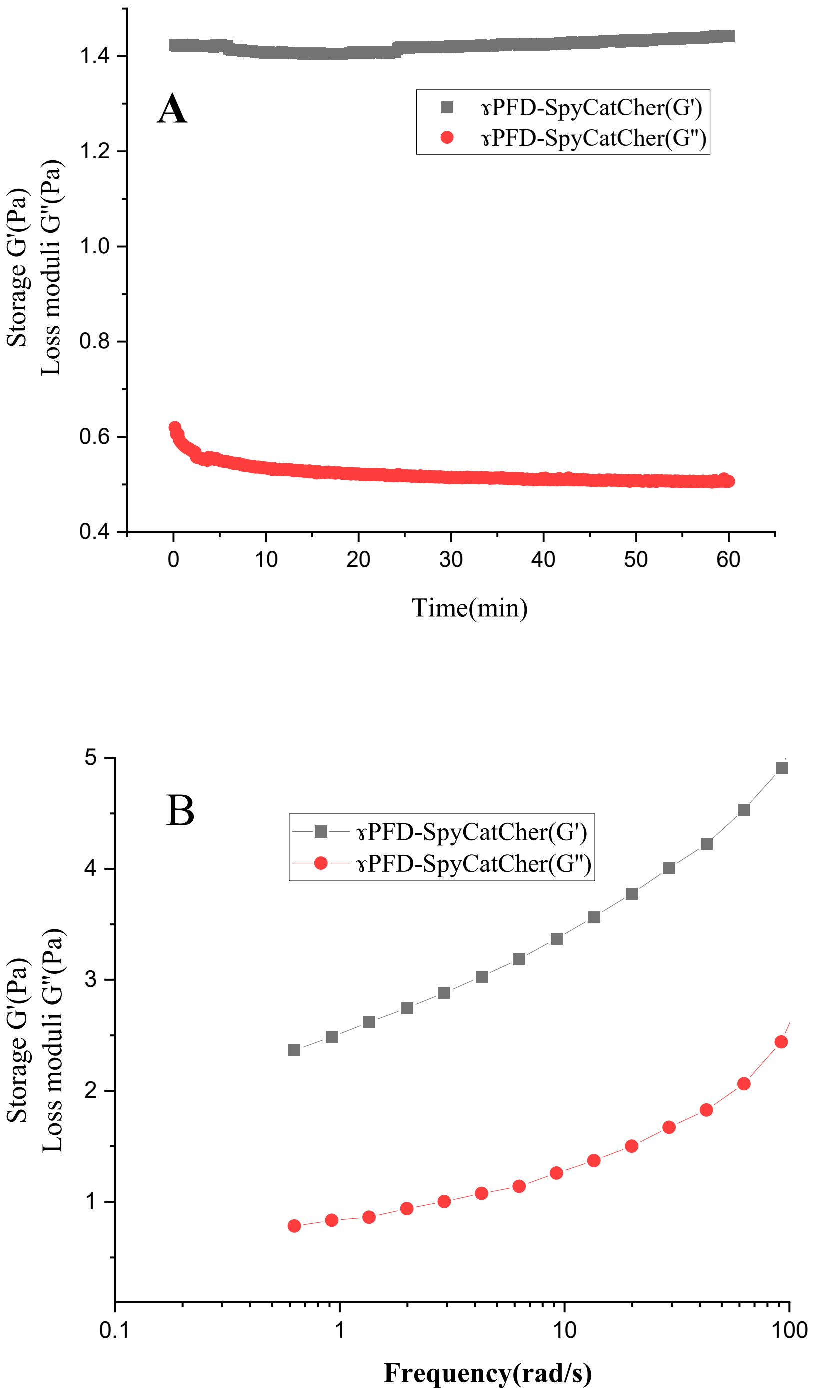
A
ɤPFD-SpyCatCher(G')
ɤPFD-SpyCatCher(G")
Storage G'(Pa)
Loss moduli G"(Pa)
1.4
1.2
1.0
0.8
0.6
0.4
0
10
20
30
40
50
60
Time(min)
B
ɤPFD-SpyCatCher(G')
ɤPFD-SpyCatCher(G")
Storage G'(Pa)
Loss moduli G"(Pa)
5
4
3
2
1
0.1
1
10
100
Frequency(rad/s)

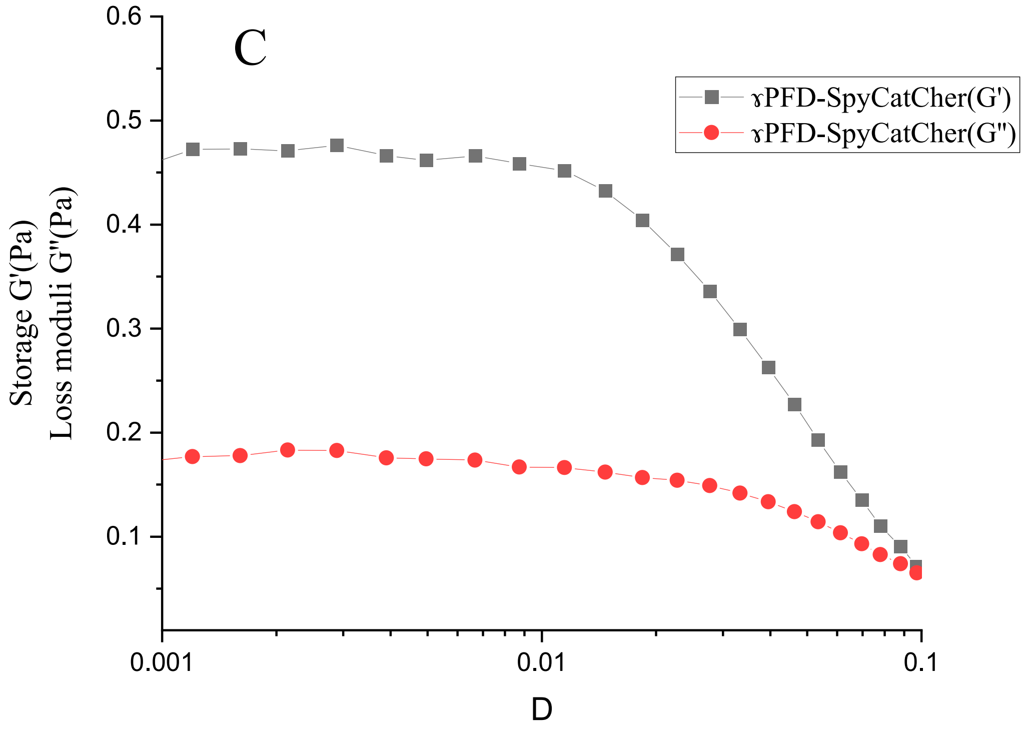

Supplementary Figure S2 SpyCatcher-mediated ɤPFD protein hydrogel viscoelastic properties

(A) Time-dependent viscoelastic moduli (G’ and G”). (B) Frequency sweep test (G’ and G”).

(C) Shear stress test (G’ and G”).

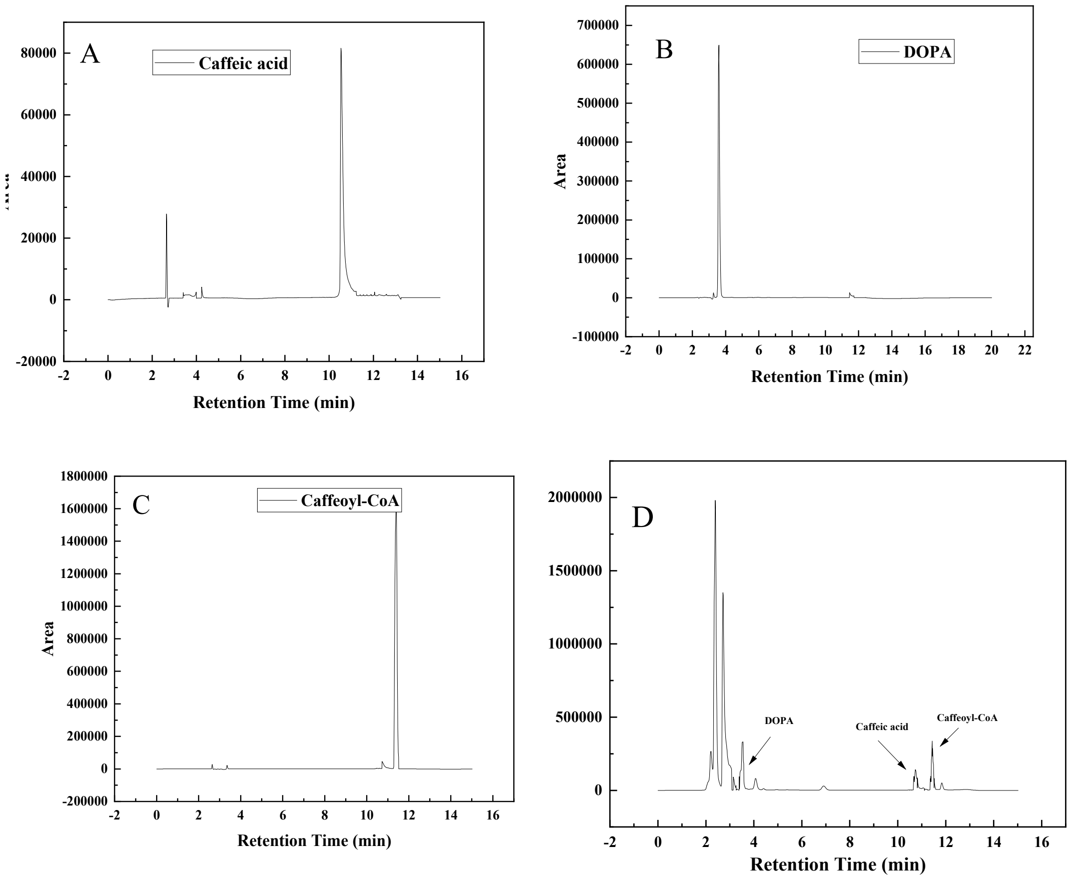

Supplementary Figure S3 HPLC chromatogram.

(A) The standard sample of caffeic acid. (B) The standard sample of DOPA. (C) The standard sample of Caffeoyl-CoA. (D) SpyTag-cycled HpaBC-TAL-4CL.

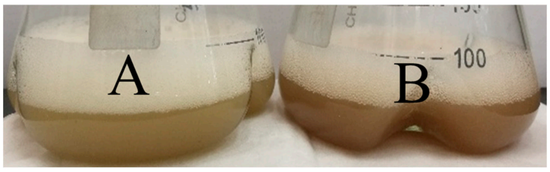

Supplementary Figure S4 Color contrast during the fermentation process.

(A) E. coli TYR containing pZEA-SpyTag- TAL- HpaBC-4CL-SpyTag and pZS-ɤPFD-SpyCatcher. (B) E. coli TYR containing pZEA-SpyTag-HpaBC-TAL-4CL-SpyTag and pZS-ɤPFD-SpyCatcher. The color of the fermentation broth can be visually assessed to indicate a significant residual DOPA based Figure 2B.
